# Insecticide resistance in *Culex quinquefasciatus *from Zanzibar: implications for vector control programmes

**DOI:** 10.1186/1756-3305-5-78

**Published:** 2012-04-21

**Authors:** Christopher M Jones, Camille Machin, Khalfan Mohammed, Silas Majambere, Abdullah S Ali, Bakari O Khatib, Juma Mcha, Hilary Ranson, Louise A Kelly-Hope

**Affiliations:** 1 Liverpool School of Tropical Medicine, Pembroke Place, Liverpool L3 5QA, UK; 2 Ministry of Health and Social Welfare, Miazini, Zanzibar, Tanzania; 3 Ifakara Health Institute, Kiko Avenue, Mikocheni, P.O. Box 78373 Dar es Salaam, Tanzania; 4 Zanzibar National Malaria Control Programme, M'kwerekwe Street, Ministry of Health and Social Welfare, Zanzibar, Tanzania

**Keywords:** Lymphatic filariasis, Malaria, Vector control, Insecticide resistance, Zanzibar

## Abstract

**Background:**

Zanzibar has a long history of lymphatic filariasis (LF) caused by the filarial parasite *Wuchereria bancrofti*, and transmitted by the mosquito *Culex quinquefasciatus *Say. The LF Programme in Zanzibar has successfully implemented mass drug administration (MDA) to interrupt transmission, and is now in the elimination phase. Monitoring infections in mosquitoes, and assessing the potential role of interventions such as vector control, is important in case the disease re-emerges as a public health problem. Here, we examine *Culex *mosquito species from the two main islands to detect *W. bancrofti *infection and to determine levels of susceptibility to the insecticides used for vector control.

**Methods:**

*Culex *mosquitoes collected during routine catches in Vitongoji, Pemba Island, and Makadara, Unguja Island were tested for *W. bancrofti *infection using PCR. Insecticide bioassays on *Culex *mosquitoes were performed to determine susceptibility to permethrin, deltamethrin, lambda-cyhalothrin, DDT and bendiocarb. Additional synergism assays with piperonyl butoxide (PBO) were used for lambda-cyhalothrin. Pyrosequencing was used to determine the *kdr *genotype and sequencing of the mitochondrial cytochrome oxidase I (mtCOI) subunit performed to identify ambiguous *Culex *species.

**Results:**

None of the wild-caught *Culex *mosquitoes analysed were found to be positive for *W. bancrofti*. High frequencies of resistance to all insecticides were found in Wete, Pemba Island, whereas *Culex *from the nearby site of Tibirinzi (Pemba) and in Kilimani, Unguja Island remained relatively susceptible. Species identification confirmed that mosquitoes from Wete were *Culex quinquefasciatus*. The majority of the *Culex *collected from Tibirinzi and all from Kilimani could not be identified to species by molecular assays. Two alternative *kdr *alleles, both resulting in a L1014F substitution were detected in *Cx. quinquefasciatus *from Wete with no homozygote susceptible detected. Metabolic resistance to pyrethroids was also implicated by PBO synergism assays.

**Conclusions:**

Results from the xenomonitoring are encouraging for the LF programme in Zanzibar. However, the high levels of pyrethroid resistance found in the principle LF vector in Pemba Island will need to be taken into consideration if vector control is to be implemented as part of the elimination programme.

## Background

The Zanzibar archipelago has a long history of lymphatic filariasis (LF), a disabling mosquito-borne disease caused by the filarial parasite *Wuchereria bancrofti *[1-6]. The mosquito species *Culex quinquefasciatus *Say, is the most important vector of *W. bancrofti *in the East African coast and the islands of the Indian Ocean, including Zanzibar, however *Anopheles gambiae *s.l and *An. funestus *also play a role in selected areas [7-10]. *Cx. quinquefasciatus *is a member of the *Culex pipiens *complex Linnaeus and one of the main subspecies found in Africa [11-13]. It is efficient at maintaining low levels of microfilariae (Mf) within a population, highly anthropophilic, and predominately bites at night. This species is a major biting nuisance, particularly in urban areas where it thrives in wet pit latrines, cess pits, blocked open drains, and polluted puddles. In Zanzibar, *W. bancrofti *Mf rates have ranged from 0.3% to 20.3% in *Culex *species [1-3,6,7,14], unpublished data.

LF has historically been a significant public health problem in Zanzibar, particularly on the main islands of Unguja and Pemba, where human *W. bancrofti *Mf rates ranged from 3% to 49%, and clinical manifestations such as hydrocele and lymphodema (elephantiasis) were common [1-6]. The LF programme was established in 1994 and a mass drug administration (MDA) campaign initiated to treat all the eligible population in 2001 [4,5] following the launch of the Global Programme to Eliminate LF (GPELF) by the World Health Organization (WHO) [15]. Zanzibar was one of the first places in the United Republic of Tanzania and sub-Saharan Africa to target LF elimination and implement six consecutive rounds of annual MDA using a combination of ivermectin and albendazole with the aim of interrupting *W. bancrofti *transmission [4,5]. It achieved >80% MDA coverage with a reduction in LF prevalence to 0% Mf rate, and has potentially reached its goal of elimination. However, with no systematic post-MDA surveillance in place it is not possible to fully determine if disease transmission has been completely interrupted. Xenomonitoring presents a cost-effective way of monitoring LF within a population [16-18] and opportunities to collect and examine mosquitoes within existing vector surveillance programmes should be utilised where possible.

Assessing the additional impact of vector control and monitoring insecticide resistance is also important in Zanzibar as there has recently been extensive scale up of indoor residual spraying (IRS) and distribution of insecticide treated/long lasting nets (ITNs/LLINs) as part of the Zanzibar Malaria Control Programme (ZMCP), supported by the Presidents Malaria Initiative (PMI) and other international donors [19,20]. Zanzibar is one of the first places in Tanzania and sub-Saharan Africa to be targeted for malaria elimination, and since 2006 has conducted five rounds of IRS with the pyrethroid insecticide lambda-cyhalothrin [19]. More than 90% IRS coverage has been achieved, protecting over one million people. Free ITNs/LLINs have also been distributed and it is estimated that > 75% of households own at least one ITN/LLIN [19]. It is possible that *Culex *populations may be affected by the wide use of insecticides and developed resistance even though these species were not being targeted. There is precedent for this in Zanzibar where previous vector control using organophosphate and organochlorine insecticides have been carried out in the 1950s-80s [21,22] and resistance in *Culex *and *Anopheles *species detected [23-26].

Widespread resistance could prove problematic if LF were to re-emerge or be re-introduced from the endemic mainland of Tanzania or Kenya [9,10], and supplementary vector control using insecticides were required in addition to MDA [15]. Insecticide resistance in *Culex *species could also impact on the malaria elimination programme if communities perceive a reduced efficacy of IRS and ITN/LLINs and usage rates decline. Given the post-MDA phase and importance of surveillance in Zanzibar, this study aimed to examine *Culex *species to detect *W. bancrofti *infection from routine entomological collections and to determine levels of susceptibility to insecticides used in vector control programmes.

## Methods

### Detection of *Wuchereria bancrofti*

**Figure 1:**
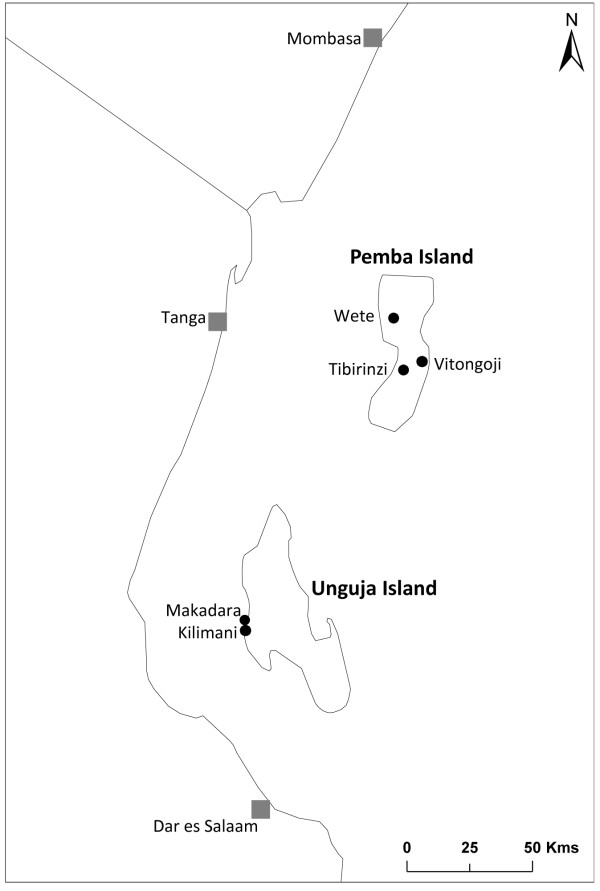
**Study sites on Unguja and Pemba Islands, Zanzibar**.

Blood-fed mosquitoes were collected from the inside of houses using pyrethroid spray catches (PSC) with permission from the village chief and the head of each household. The PSCs are part of routine entomological monitoring by the ZMCP which are carried out every 2 to 4 weeks and collections were made between 0530 and 0930 hours during April-May 2011. For the purpose of this study, only female culicines were used with the remaining males being discarded. Collections were conducted from the following sites: Vitongoji (Pemba) 5 °12'42" South, 39 °49'51" East, Makadara (Unguja) 6 °09'93" South, 39 °11'54" East (Figure 1).

Genomic DNA (gDNA) was extracted from 226 individual mosquitoes collected from the PSCs using the DNeasy kit (QIAGEN) following the manufacturer's instructions. The presence or absence of *W. bancrofti *in *Culex *mosquitoes was detected using the PCR diagnostic described by Ramzy *et al*. [27]. gDNA from five individual mosquitoes were pooled according to the respective sites and screened. If the pooled sample yielded a positive result, mosquitoes from that pool were screened to isolate the *W. bancrofti*-positive individual. To confirm any putative positive samples, a melt-curve analysis was conducted using the diagnostic primers from Ramzy *et al*. [27]. Melt curve analysis is a quantitative PCR (qPCR) assay which determines the specificity of amplified PCR products according to their unique melting temperatures (T_m_). The 188 bp amplified product was detected using the Brilliant III Ultra-Fast SYBR^® ^Green Master Mix (Agilent Technologies) on the Stratagene Mx3005P (Agilent Technologies). Real-time PCR reactions were run following a thermal profile of 3 mins at 95°C followed by 40 cycles of 10 s at 95°C and 10 s at 60°C. Immediately following amplification, melt curves were generated by increasing the temperature incrementally from 55°C to 95°C while SYBR^® ^Green is continually detected. The presence of a single homogeneous peak in the melt curve plot (temperature versus first derivative of raw fluorescence) is indicative of specific amplification (characteristic T_m_).

### *Culex* insecticide susceptibility assays

*Culex *larvae (L1 to L4) were collected from two sites on Pemba (Wete: 5 °3'21" South, 39 °43'45" East; Tibirinzi: 5 °14'31" South, 39 °45'53" East) and from one site on Unguja (Kilimani - 6 °10'5" South, 39 °12'49" East) (Figure 1). All larvae were transported to the insectaries of ZMCP for adult rearing.

Approximately one-hundred non-blood-fed female 2-5 day old adults were exposed to the following WHO insecticide-treated papers for one-hour: permethrin (0.75%), deltamethrin (0.05%), lambda-cyhalothrin (0.05%), DDT (4%) and bendiocarb (0.1%). The percentage mosquito mortality was recorded 24 hours later. Control assays were performed throughout the experiment with a minimum of 25 mosquitoes exposed to non-insecticide treated papers.

In order to determine the level of lambda-cyhalothrin resistance, time-mortality response assays were conducted from Wete (Pemba) and Kilimani (Unguja). Approximately one-hundred *Culex *mosquitoes were exposed to lambda-cyhalothrin (0.05%) treated papers for six time points from Wete (30, 60, 90, 120, 240 and 360 mins) and four time points from Kilimani (15, 30, 45 and 60 mins). Control assays using non-insecticide treated papers were conducted throughout.

### PBO synergist assays

To investigate the potential involvement of metabolic resistance in *Culex *from Wete, mosquitoes were pre-exposed to the synergist PBO (piperonyl butoxide); a known inhibitor of P450 and esterase activity. Approximately 75 female mosquitoes were pre-exposed to 4% PBO-treated papers for one-hour and immediately exposed to lambda-cyhalothrin (0.05%) for a further hour. Mortality was scored 24 hours later and any synergism compared with mortality from assays conducted without pre-exposure to PBO (described above).

### PCR identification of *Culex quinquefasciatus*

*Cx. quinquefasciatus *is a member of the *Cx. pipiens *species complex and is generally assumed to predominate *Culex *mosquitoes from Zanzibar [10-14]. The PCR diagnostic described by Smith and Fonseca [28] uses a series of diagnostic primers to discriminate four members of the *Culex pipiens *complex plus *Cx. torrentium *and *Cx. pervigilans *based on single nucleotide polymorphisms (SNPs) in an intron of the acetylcholinesterase-2 (*ace-2*) gene. The primers *ACEquin *(5'-CCTTCTTGAATGGCTGTGGCA-3') and *B1246 *(5'-TGGAGCCTCCTCTTCACGG-3') amplify a 274 bp diagnostic fragment of *Cx. quinquefasciatus*. In this study, 1 μl of gDNA from 19-24 individuals from Wete (Pemba), Tibirinzi (Pemba) and Kilimani (Unguja) were added to 20 μl PCR reactions using the *Cx. quinquefasciatus *specific primers under the following thermal cycling conditions: 95°C for 2 mins, followed by 35 cycles of 95°C for 30 s, 55°C for 30 s and 72°C for 16 s with a final extension step of 72°C for 2 mins. Individuals which failed to amplify after two attempts were classed as 'other' species.

To confirm the presence of additional *Culex *species on Zanzibar, 1 μl of gDNA from a subset of *Cx. quinquefasciatus *positive and negative samples was used to amplify a ~800 bp region of the mitochondrial cytochrome oxidase I (mtCOI) sequence. mtCOI is a common molecular marker for cryptic species complexes in insects and the universal primers used to amplify this region have been described elsewhere [29]. The PCR product was purified (QIAquick PCR-Purification kit; QIAGEN) and sequenced in both the forward and reverse direction using the reaction PCR primers by Macrogen Inc. (Amsterdam, Netherlands). Sequences were analysed and aligned in CodonCode Aligner (CodonCode Corporation, Dedham, MA) and compared with other Culicine mtCOI sequences from an independent study at LSTM (D. Weetman pers. communication). Unique sequences were submitted to GenBank.

### Target-site resistance mutations

Genomic DNA was extracted from mosquitoes exposed to lamda-cyhalothrin (0.05%) for 240 mins from Wete using either the DNeasy extraction kit (QIAGEN) or the 'Livak' protocol described previously [30]. For the Livak method, individual mosquitoes were homogenised in 100 μl of pre-heated (65°C) Livak buffer [30] and samples incubated at 65°C for 30 min. Potassium acetate was added (14 μl of 8 M stock) and the samples incubated on ice for 30 min. The supernatant was collected following centrifugation (13,200 rpm for 20 min) and mixed with 100% ethanol at 13,200 rpm for 15 min. The DNA pellet was washed in 70% ethanol, air-dried for 1 hr and re-suspended in 100 μl of sterile distilled water.

A pyrosequencing assay was used to determine the genotype at position 1014 (*kdr *site) in the sodium channel [31]. The assay detects one of three potential nucleotides (A/T/C) at the third position in the 1014 codon [31]. In brief, a 154 bp region was PCR-amplified with forward and biotinylated reverse primers (Table 1). The sequence analysed to detect the genotype at 1014 was 5'-TT[A/C/T]GTCGTGAGTATTCCAG-3'. Pyrosequencing reactions were performed using the PyroMark Gold Q96 Reagents Kit (QIAGEN) on the PyroMark Q96 system (QIAGEN). The relative heights of the bioluminescence peaks for each nucleotide were used to genotype each individual [31].

**Table 1:** Detection of *W. bancrofti *in *Culex *spp. caught from PSC in Zanzibar

		*W. bancrofti *detection*	
**Island**	**Site**	**Positive**	**Negative**

Pemba	Vitongoji	0	150

Unguja	Makadara	0	76

*Presence or absence of *W. bancrofti *determined using PCR diagnostic described Ramzy *et al*. (1997)

Resistance to carbamates and organophosphates in *Anopheles *and *Culex *mosquitoes is associated with a mutation (glycine to serine at position 119 or G119S) in the *ace-1 *gene encoding acetylcholinesterase (AChE) [32]. The presence of *G119S *in *Culex *mosquitoes from Zanzibar was determined using the Restriction Fragment Length Polymorphism (RFLP) assay described by Weill *et al*. [33]. In brief, gDNA from 20 individuals from Wete (Pemba) and Kilimani (Unguja) was added to a 20 μl PCR reaction using the degenerate Moustdir1 and Moustrev1 primers which amplify a 194 bp fragment of *ace-1*. PCR products were digested with the *Alu*I restriction enzyme and run on a 1.5% agarose gel.

### Statistical analysis

Exact 95% confidence intervals for knockdown and mortality data from discriminatory dose bioassays were calculated in R for Windows version 2.2. Time response curves and LT_50 _and LT_90 _values were generated using Probit analysis in XL STAT.

## Results

### Detection of *W. bancrofti* from Zanzibar

**Figure 2:**
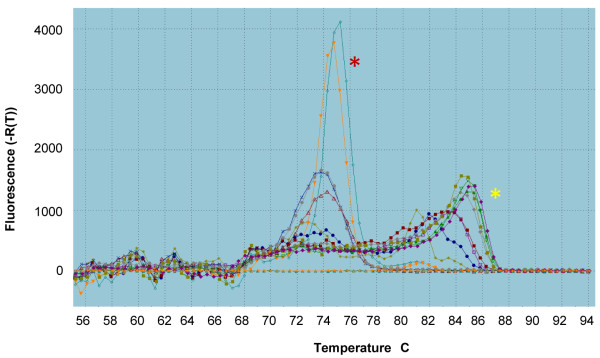
**Melt-curve profile for determining the presence or absence of *W. bancrofti *in *Culex *mosquitoes. **The red asterix adjacent to specific peaks in the melt curve indicates *W. bancrofti *infected *Culex *(T_m _= 74.3-74.8). The yellow asterix adjacent to non-specific peaks in the melt curve profile indicates *Culex *mosquitoes free from *W. bancrofti *infection.

A total of 226 mosquitoes caught by PSC from Zanzibar (150 from Vitongoji (Pemba) and 76 from Makadara (Unguja) were examined for the presence of *W. bancrofti *(Table 1). None of the wild-caught *Culex *mosquitoes analysed were found to be positive for *W. bancrofti*. In a separate study conducted in parallel with the data presented here, *W. bancrofti *infected *Culex *were identified from Dar es Salaam by both the standard PCR diagnostic [27] and melt-curve analysis (Figure 2), confirming that the sensitivity of both assays is sufficiently reliable for detecting *W. bancrofti *from wild-caught mosquitoes.

### Insecticide resistance bioassays

**Figure 3:**
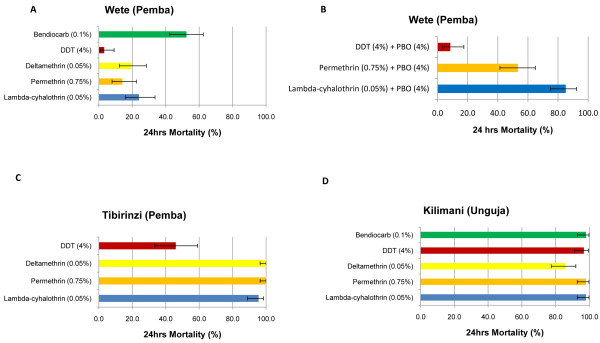
**Percentage mortality of *Culex *mosquitoes from Zanzibar following exposure to WHO insecticide treated papers**. Percentage mortality and 95% confidence intervals from a) Wete (Pemba) b) Wete (Pemba) with pre-exposure to PBO (4%) c) Tibirinzi (Pemba) d) Kilimani (Unguja).

*Culex *mosquitoes from Wete (Pemba) were resistant to all insecticides tested (Figure 3a). Low mortality levels were obtained after exposure of *Culex *from Wete to pyrethroid or DDT (deltamethrin = 19.4%, 95% CI: 12.3-28.4%; permethrin = 14.0%, 95% CI: 7.9-22.4%; lambda-cyhalothrin = 24.0%, 95% CI: 16.0-33.6%; DDT = 3.2%, 95% CI: 0.7-9.1%). Moderate mortality rates were observed after exposure to the carbamate, bendiocarb (52.4%, 95% CI: 42.4%-62.4%). In contrast to Wete, mosquitoes from the other site tested on Pemba, Tibirinzi, were almost fully susceptible to all three pyrethroids (Figure 3c). Although resistance to DDT was detected from Tibirinzi, this was at a lower level than Wete (46.0%, 95% CI: 33.4-59.1). *Culex *populations from Unguja were largely susceptible to all insecticides with only deltamethrin giving any detectable level of resistance (86% mortality, 95% CI: 77.4-92.0%) (Figure 3d).

Insecticide resistant mosquitoes from Wete on Pemba were pre-exposed to the synergist PBO (4%) for one-hour before exposure to lambda-cyhalothrin (0.05%), permethrin (0.75%) and DDT (4%). Synergism was evident against the pyrethroids with mortality rising to 53.3% and 85.1% for permethrin and lambda-cyhalothrin respectively while there was no evidence of synergism with DDT (8.5%, 95% CI: 3.2-17.5%) (Figure 3b). This suggests that metabolic (e.g. P450-mediated) resistance may contribute to the resistance phenotype but is not the sole mechanism in *Culex *from Wete.

Assays to determine the LT_50 _(lethal time taken to kill approximately 50% of mosquitoes) for lambda-cyhalothrin were performed for *Culex *from Wete and Kilimani. Generation of probit curves for Kilimani *Culex *were rejected due to the relatively high levels of susceptibility to lambda-cyhalothrin in this population (P > 0.05). However, the estimated LT_50 _for the Wete population was 199.4 mins (95 CI % = 183.3 - 217.8 mins), demonstrating the extremely high levels of resistance to this pyrethroid.

### Molecular determination of *Culex *species

**Figure 4:**
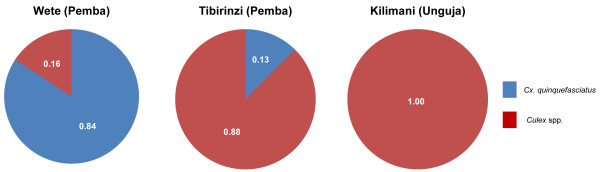
**The frequency of *Cx. quinquefasciatus *from the study sites on Zanzibar. ***Cx. quinquefasciatus *specific primers were used to discriminate this species from samples from Wete (N = 19), Tibirinzi (N = 24) and Kilimani (N = 20). Samples which failed to amplify were classed as unknown '*Culex *spp'.

Confirmation of *Cx. quinquefasciatus *from each of the insecticide resistance study sites on Zanzibar was performed using the method described by Smith and Fonseca [28]. Eighty-four percent of *Culex *from Wete on Pemba Island (N = 19) were confirmed as *Cx. quinquefasciatus*. In contrast, only 13% and 0% of the mosquitoes from Tibirinzi (Pemba) (N = 24) and Kilimani (Unguja) (N = 20) respectively were identified as *Cx. quinquefasciatus *using this molecular diagnostic (Figure 4). A 760 bp region of the mtCOI marker from a sub-set of *Cx. quinquefasciatus *and unidentified *Culex *spp. was therefore compared with other available Culicine sequences. All samples sequenced from Wete had a single mtCOI haplotype (GenBank acc. no. JN990140), which was consistent with other members of the *Cx. pipiens *species complex. Seventy-one polymorphic sites existed between the population from Wete and four distinct haplotypes that were found in Tibirinzi (GenBank acc. no's JN990141-JN990144). The Tibirinzi haplotypes clustered into a separate group when compared with other Culicine mtCOI sequences (D. Weetman pers. communication) and therefore could not be identified to species level.

### Frequency of target-site resistance mutations

Mosquitoes from Wete were collected either (i) following exposure to lambda-cyhalothrin (0.05%) for 240 minutes (dead or alive) or (ii) without exposure to insecticide treatment, for genotyping at the 1014 *kdr *position in the voltage-gated sodium channel. The wild-type allele (TTA) was absent from all samples genotyped using the pyrosequencing assay. Two variant substitutions at the third coding position of 1014 were identified, both of which result in an amino acid change from leucine to phenylalanine (L1014F), TTT and TTC. Homozygous T/T individuals predominated in this population (genotype frequency = 0.73) with heterozygote T/C and homozygous C/C at lower levels (genotype frequencies = 0.23 and 0.04 respectively). However, since all genotypes encode phenylalanine at 1014, the alternative codons are unlikely to have any impact on the phenotype. Indeed, there was no apparent difference in the frequency of each genotype between survivors and dead from insecticide treatment or between those unexposed (Table 2).

**Table 2:** Frequency of 1014 *kdr *genotypes in *Culex *mosquitoes from Wete with or without exposure to lambda-cyhalothrin (0

					Genotype*		
**Treatment**	**Phenotype**	**N**	**A/A**	**A/T**	**T/T**	**C/T**	**C/C**

Lambda-cyhalothrin (0.05%)^λ^	Dead	26	0	0	15 (0.58)	11 (0.42)	0

	Alive	29	0	0	26 (0.90)	3 (0.10)	0

Unexposed	Control	14	0	0	9 (0.64)	2 (0.14)	3 (0.21)

	Total	69	0	0	50 (0.73)	16 (0.23)	3 (0.04)

*1014 genotype at the third coding position determined using pyrosequencing

^λ^Following 240 minutes exposure to lambda-cyhalothrin (0.05%)

Twenty *Culex *mosquitoes were screened for the presence of the G119S mutation in the *ace-1 *gene from Wete and Kilimani. Three heterozygous individuals were detected from Wete (frequency of 119 S = 0.08) while no resistant genotypes were found in the samples screened from Kilimani suggesting insensitive acetylcholinesterase may play a partial role in the carbamate resistance observed from Wete.

## Discussion

This study examining *W. bancrofti *infection in *Culex *species from the two main islands of Zanzibar in the post-MDA phase of the LF Programme (now incorporated into the Neglected Tropical Disease (NTD) Programme) [5], provides valuable information on the main vector. However, to fully confirm the interruption of LF transmission, it would be necessary to increase sample sizes significantly, and extend xenomonitoring activities over time and space in accordance with current recommendations [18], and with the support of national entomologists, scientists and vector control programmes.

Synergies between LF and malaria vector control activities are encouraged by WHO [34], and the Zanzibar NTD Programme is well placed to take advantage of the routine surveillance carried out for malaria by the ZMCP, where thousands of *Culex *mosquitoes are collected at sentinel sites each year. Systemic testing of *W. bancrofti *in *Cx. quinquefasciatus *may provide early warning signs of recrudescence or re-introduction from the endemic mainland where human travel is frequent [35], especially as there is evidence that antigen carriers may have re-emerged on the islands and PCR positive mosquitoes have been detected in other locations in recent years (unpublished observations). Systematic surveillance could help to target further interventions if required, including vector control.

The extent to which IRS and ITN/LLIN activities for malaria have played a role in reducing LF prevalence or maintaining <25% Mf rates in Zanzibar is unclear. However, the concurrent scale up of malaria vector control with the sixth and final round of MDA for LF may well have increased the impact on *W. bancrofti *transmission at the time [19,20,36,37]. The use of IRS and ITN/LLINs in other countries in Africa [38-40] and the Pacific [41-43] have been shown to be effective in reducing LF prevalence, however transmission was by *Anopheles *vectors, and predominately in rural areas.

Effective vector control of *Cx. quinquefasciatus *in urban areas of Tanzania has been achieved through environmental improvement, larval source reduction, application of polystyrene beads and use of insecticides [8,11,44-48]. For example, previous studies in Zanzibar have shown significant reductions of *W. bancrofti *infection rates in both human and *Cx. quinquefasciatus *by using polystyrene beads in wet pit latrines [7]. In Zanzibar and Dar es Salaam, larviciding with chlorpyrifos was used effectively in flooded pit latrine and septic tanks during the 1970s-80s, however resistance to this insecticide was detected after a number of years [23,24,44,49]. None of these methods are currently being employed in Zanzibar.

This is the first study we are aware of to document insecticide resistance levels in *Culex *species from Zanzibar in the last three decades. Organophosphate resistance has been previously reported in *Cx. quinquefasciatus *from Zanzibar [23,24], and pyrethroid, organophosphate and carbamate resistance in *Cx. quinquefasciatus *from Tanzania mainland [44,50,51]. Here, strong resistance to all insecticide classes tested were found in *Cx. quinquefasciatus *from Wete on Pemba Island, whereas relative susceptibility was found in the nearby site of Tibirinzi (Pemba) and in Kilimani on Unguja Island. Comparing resistance between these sites is not possible given the unexpected finding that the majority of *Culex *caught from Tibirinzi and Kilimani are apparently not members of the *Cx. quinquefasciatus *complex. Unfortunately, morphological identification was not performed on these species and hence their identity and behavioural patterns remain unknown. It is possible that these species do not enter houses, and are thus not affected by IRS, which may explain their lack of resistance.

The high levels of resistance in *Cx. quinquefasciatus *may be related to the current malaria control activities in Zanzibar. Two variants of the *kdr *1014 F allele were detected from all *Culex *sampled from Pemba, both of which result in a leucine to phenylalanine substitution at codon 1014. This substitution is strongly associated with pyrethroid and DDT resistance in *An. gambiae *s.l. [52]. The A-to-T mutation has been reported from different members of the *Cx. pipiens *complex including pyrethroid resistant *Cx. quinquefasciatus *from West and East Africa [50,53-55]. The second mutation, an A-to-C substitution, was found at a lower frequency from Wete and has been previously documented from *Cx. quinquefasciatus *from Sri Lanka [31] but not from the African continent. The fixation of 1014 F in the Wete population and cross resistance with DDT, suggests that selection pressures have been acting against *kdr *for some time.

The historical and extensive use of DDT in IRS during the 1950s-80s [21,22], local agricultural usages and/or extensive use of pyrethroids in malaria control [52,56], could all feasibly play a role in selecting for *kdr *in *Culex *from Pemba. The synergism with PBO however, indicates that other mechanisms, such as P450-mediated detoxification, may also be contributing to the observed resistance patterns. Whatever the mechanisms involved, the strong levels of pyrethroid resistance in *Cx. quinquefasciatus *from Wete (LT_50 _= 199.4 mins) could have broader implications for the control of all vector-borne diseases on Pemba Island, particularly if it reduces community engagement in vector control programmes. Furthermore, the presence of bendiocarb resistance (52.4% mortality) together with 119 S in *ace-1 *in Wete, albeit at a low frequency, means that switching to a carbamate-based vector control strategy, may not extensively suppress *Culex *from this area and indeed, potentially select for further resistance.

## Conclusion

Insecticide resistance in *Cx. quinquefasciatus *is of concern for the NTD Programme if insecticide-based control methods are considered as an intervention in the future. However, this is unlikely in this setting given the effectiveness of the current drugs used for elimination [15]. Moreover, further rounds of MDA in combination with alternative vector control methods such as polystyrene beads may be more effective and practical if LF were to re-emerge as a public health problem in Zanzibar.

## Competing interests

The authors declare that they have no competing interests.

## Authors' contributions

LKH and HR conceived the study. CM, CMJ, KM, SM, BK, JM and AA carried out the field work. CM, CMJ and HR designed and performed the lab experiments. CM, CMJ, HR, and LKH analyzed the data, interpreted the results and wrote the first draft of the paper. All authors read and approved the final manuscript.
